# Organizational facilitators and barriers for participation in workplace health promotion in healthcare: A qualitative interview study among nurses

**DOI:** 10.3389/fpsyg.2023.1101235

**Published:** 2023-03-02

**Authors:** Hannah Bleier, Jasmin Lützerath, Andrea Schaller

**Affiliations:** ^1^Working Group Physical Activity-Related Prevention Research, Institute of Movement Therapy and Movement-oriented Prevention and Rehabilitation, German Sport University Cologne, Cologne, Germany; ^2^Institute for Workplace Health Promotion, Cologne, Germany

**Keywords:** workplace health promotion, participation, nurses, barrier, WHP activity, organizational framework conditions, facilitator

## Abstract

**Background:**

There is evidence for the positive effects of workplace health promotion (WHP) for nurses. Although this highly stressed target group also actively desires WHP, the number of participants is low. Individual reasons play a role in the decision to engage in WHP activities, yet it is interesting to consider which organizational factors a company could address to improve participation. In this regard, the question arises of what organizational factors facilitate participation in WHP activities from the perspective of nurses in inpatient care facility (ICF), outpatient care service (OCS), and acute care hospitals (ACH).

**Method:**

Sixteen semi-structured interviews were conducted in different care settings between May and September 2021. Questions about everyday working life, WHP activities, and organizational framework conditions were asked.

**Result:**

The results show that there is a wide range of influencing factors at the organizational level, some overall settings, and others setting-specific. High workload and the fit of WHP activities with shift times were particularly inhibiting overall settings. A negative association with the employer worked as a barrier in ICF and ACH.

**Conclusion:**

When implementing WHP activities, it can be useful to consider organizational facilitators and barriers to promote sustainable and attractive WHP activities and higher participation rates in the different settings of nursing.

## 1. Introduction

Workplace health promotion is a promising approach on policy, company, and individual levels to counteract work-related stress and make jobs attractive. There are indications that workplace health promotion (WHP) can improve the health and work ability of employees (Hupfeld et al., 2021). The Luxembourg Declaration defines WHP as the joint action of society and workplace to improve the health of employees (the European Network for Workplace Health Promotion set itself the task of supporting employers, employees, and society in safeguarding and promoting wellbeing and health in the workplace through the Luxembourg Declaration). Improvements can be made here through the enhancement of work organization and environment, improving active participation, and supporting personal development (European Network for Workplace Health, 1997). Especially for professions with high occupational stress (high mental and physical workload), such as nurses (Meyer et al., 2022), WHP, therefore, can be a promising approach to improve the work situation and personal health. Among companies and statutory health insurers, there is a strong collaborative commitment to WHP because jobs in this sector are characterized by significant physical and mental stress (Bauer et al., 2020). Nurses in geriatric care in Germany, for example, were sick for 28.5 days on average in 2021. This is almost 10 days more than the average number of sick days for German employees in 2021 (19.7 days; Meyer et al., 2022). The sickness notifications of health insurance show mainly disease in the area of musculoskeletal disorders, mental illnesses, and respiratory diseases (Drupp and Meyer, 2020). However, it is not possible to deduce from this whether this is due to working conditions or a consequence of stress.

Looking at the effectiveness of WHP across all occupational groups, there are indications that WHP can be a worthwhile use of resources for employers and employees to improve work-related health. Positive effects could be demonstrated for 68.6% of the behavioral-and environmental-related prevention interventions analyzed in a narrative review (Goldgruber and Ahrens, 2009). A Cochrane review indicates that programs using pedometers can reduce the body mass index (Freak-Poli et al., 2013). In two reviews, small to medium effects are reported for the efficacy of environmental-related activities to improve the *nutritional behavior* of employees with regard to diet-related outcomes such as fruit and vegetable consumption (Geaney et al., 2013; Allan et al., 2017). For *nicotine and tobacco* use, a Cochrane review indicates clear evidence of effectiveness for group therapy approaches in the work context, personal individual counseling, drug treatment, and combined interventions (Cahill and Lancaster, 2014). Short-term *alcohol* prevention interventions seem to have potential in a review, but there is still a considerable need for research (Schulte et al., 2014). Interventions that work with cognitive behavioral therapy approaches and combine more than one therapeutic approach (for example, cognitive behavioral therapy and teaching problem-solving strategies) appear to be effective in relieving stress and improving relaxation in a review (Wan Mohd Yunus et al., 2018). Another meta-analysis, based on a total of eight qualitatively convincing RCTs, finds small but nonetheless significant positive effects for *depression prevention* interventions at the whole-firm level (Hosang et al., 2014). With regard to the benefit for employers, a comprehensive review of 47 return-on-invest (ROI) studies on WHP shows a mean ROI of 2.7 (Baxter et al., 2014). In addition to the interest of employees and indications of a benefit for employers, health insurance funds are also pursuing this approach. Having a look at the effect of WHP activities on nurses, only a very limited amount of studies are published (Schaller et al., 2022). Yet, the compilation of studies on lifestyle-based, health-promoting interventions for nurses consistently reports positive effects on outcomes such as physical activity behaviors, mobility, and endurance (Chan and Perry, 2012). A meta-analysis based on available RCTs for mindfulness-based interventions in nurses suggests that the interventions are suitable for reducing anxiety and depression in the short and long term in this target group. Qualitative studies also indicate other positive aspects of impact, including improved wellbeing (e.g., increased inner calm) and increased work performance (e.g., more efficient work processes due to improved team communication; Guillaumie et al., 2017). A quantitative systematic review of interventions for promoting nurses’ wellbeing at work suggests that the interventions can also achieve lasting effects (Romppanen and Häggman-Laitila, 2017).

Despite the burden on nurses and the indications of positive effects, nurses have lower participation rates in WHP activities than other professions (Chiou et al., 2014). Furthermore, a study on German nurses shows a self-reported willingness of 75% to actually use a prevention program (Ehegartner et al., 2020). More than every second facility manager reports that existing WHP activities are only insufficiently used (Isfort et al., 2018). In addition to individual reasons for non-participation, it might be promising to consider the organizational factors a company can address in order to increase the uptake of WHP activities among employees (Rojatz et al., 2015).

Having a look at organizational factors influencing participation in WHP activities overall professions, there are some findings. In this context, a lack of management support, a lack of qualified trainers, related costs, lack of space, and evidence of program outcomes were reported as potential challenges over all professions in the United States (Weinstein and Cheddie, 2021). Furthermore, a systematic review of countries in the “Organisation for Economic Co-operation and Development” (OECD) indicates that the working environment and organizational structures, management support for the activity, and the coordination of the activity with existing structures and processes can play a role in the success of WHP projects (Rojatz et al., 2015). Incentives and leadership support can significantly impact participation. Higher levels of organizational support were also shown to raise better participation in biometric screening and health assessment (Grossmeier et al., 2020). The quality of the intervention concept and the material and resources of the target group can make a matter as well (Rojatz et al., 2015). Across the process, continuously raising awareness, participation and empowerment of employees, as well as regular internal communication can be key success factors in terms of acceptance and sustainability of WHP activities (Bauer et al., 2020). If the information is not provided clearly and unambiguously, impersonally, or at an inappropriate time, this can also have an influence on participation (Stummer et al., 2008). If the WHP activity is experienced as mandatory, this is also perceived rather negatively and is more likely to be rejected (Stummer et al., 2008). Other perceived barriers are the cost to the target group, the fact that participation causes absenteeism, and the inconvenient timing of the offerings (Simek et al., 2014). Lack of target group orientation, possible motivation problems, and general project management recommendations can also have an influence (Weinreich and Weigl, 2002). Lack of participation autonomy, lack of professionalism in the activity, and missing sense can also affect the interest in the WHP activity (Stummer et al., 2008).

In the setting of nursing, there are fewer results on organizational factors influencing participation in WHP activities. Limited time resources of nurses due to the fulfillment of the patient care mission under conditions of staff shortage seems to make it harder to make time free for participation in WHP (Krupp et al., 2020). Results of a German study in the nursing setting indicate that counting the WHP activity time spent as working time and having the employer cover the costs can have an influence on participation in WHP activities (Dietrich et al., 2015). Furthermore, from the perspective of many nurses, it does not seem consistent or “honestly meant” by the employer to offer behavioral preventive WHP activities, but at the same time not be able to reduce the burden in the everyday workday (Krupp et al., 2020). Factors such as the small size of the company (short distances, personal ties, and direct contact), lack of economic pressure, personal approach, and opportunities for participation seem to have a positive effect on participation in WHP in outpatient care service (Kahnt et al., 2020).

If one examines the field of nursing, the setting-specific differences can also become an interesting point. There are findings that WHP activities are predominantly available in large facilities such as hospitals and are often not accessible to staff in medium-sized nursing homes or outpatient care facilities (BKK Dachverband, 2017).

Regarding the little research in the field of nursing on organizational factors influencing the participation of employees in WHP activities and even less evidence on how this is perceived from the perspective of nurses, it would be interesting to investigate this topic. As organizational factors can be adjusted by companies, findings in this area can help to design activities in such a way that they improve the chances of increasing the participation rate and satisfaction among nurses.

In regard to this research gap, we investigate the following research question: *What organizational factors facilitate participation in WHP activities from the perspective of nurses in different settings?*

## 2. Materials and methods

### 2.1. Context of the study

The interviews were conducted within the BAGGer project (workplace activities for health promotion and violence prevention, 2020–2022). The aim of the project, which was funded by the German Federal Ministry of Health (BMG), was to promote health and improve the working situation of nurses. In order to understand why or why not nurses participate in WHP activities, the following study aims to find out which facilitators and barriers can be found in different settings of nursing. The study was registered in the German Clinical Trials Register (DRKS-ID: DRKS00024961) and approved by the German ethics committee of the German Sport University Cologne (reference numbers No. 050/2021). In this qualitative study, we followed the consolidated criteria for reporting qualitative research (COREQ; Tong et al., 2007).

### 2.2. Participants

Participants were recruited *via* the care facilities participating in the BAGGer project, which were located in North Rhine-Westphalia, Germany. Inclusion criteria for participants were: (1) a professional nurse, (2) minimum age of 18 years, and (3) working in an acute care hospital, inpatient care facility, or outpatient care service. Exclusion criteria were: (1) professional nurses mainly assigned to administrative working tasks and (2) apprentices. The person in charge of employee health provided information within the care facilities about the search for interview participants and acted as a contact person. Interested nurses could then get in touch with this contact person so that a random composition of the sample was generated. In the first contact between the research team and the participants, the participants were informed by email about the background of the study and aspects of data protection. In addition, an interview date was arranged by the interviewers (JL, HB) by email or telephone. All the requested persons participated and gave their written consent.

The sample comprised 16 participants (four men and 12 women). Ages ranged from 25 to 54 years (mean 39 ± 11 years). The average experience in nursing was 14 years (±9 years) with a minimum of 4 years and a maximum of 36 years. Three nurses had an immigrant background. Interview duration ranged from 36 to 171 min (mean 78 ± 33 min).

The sample composition of participants per setting is presented in Table 1.

**Table 1:** Table showing sample description and interview length per setting.

	Acute care hospital (*n* = 5)	Inpatient care facility (*n* = 6)	Outpatient care service (*n* = 5)
Age [years] mean^a^ (±SD); minimum-maximum	33 (±6); 27–42	34 (±11); 25–52	51 (±3); 47–54
Gender: female [n; %]	3 (60%)	4 (67%)	5 (100%)
Experience in the care sector [years] mean (±SD); minimum-maximum	12 (±3); 4–16	11 (±7); 5–20	20 (±12); 4–36
Length of employment at current facility [years] mean (±SD); minimum-maximum	9 (±4); 4–15	7 (±6) 0,2–15	3 (±2) 1–6
Interview duration [min] mean (±SD); minimum–maximum	69 (±7); 59–87	63 (±30); 36–119	104 (±42); 58–171

SD, Standard deviation; *n*, number.

a The average age of geriatric nurses in Germany is 43.2 years, while the average age of healthcare and nursing staff is 41.6 years (Destatis Statistisches Bundesamt, 2018).

### 2.3. Interview guide

A problem-centered interview guide based on Witzel (2000) consisting of open-end questions was developed. Questions on the topics of career, everyday work, health, workplace violence, and company were collected and collaboratively formulated by the research team. As organizational barriers and facilitators to WHP can lie in a wide variety of areas of everyday work, we decided to ask questions broadly. The topics and their content aspects are presented in Table 2. Comprehensibility and estimated interview duration were the first pilots tested in our internal project group and the second tested with a nurse working in an acute care hospital. As there were limited resources in our study and the internal as well as external pilot studies showed that the questions were easy to understand, there was no need for further pretesting. The pilot test data were not included in the main study.

**Table 2:** Topics and content aspects of the interview guide.

Topic	Content aspects
Everyday working life	- Influence of everyday working life on participation in WHP activities
Health	- Existing WHP activities in the company - Reasons for participation - Appropriate content and social design of WHP activities - Anchoring of health in the corporate culture and communication - Importance of WHP in the company - Commitment of the management to the topic - Communication of health-promoting activities and changes
Workplace violence	Prevention, trainings and support programmes for violence prevention
Company	Perception of the employer

### 2.4. Data collection

Interviews were conducted between May and September 2021 and took an average of 78 min (ranging from 36 to 171 min). At the beginning of the interview, the participants were told that ~1 h would be estimated for the interview and they could take a break if they wanted to. The spoken language was German. The interviews and pilot tests were conducted by telephone to minimize the number of contacts because of the COVID-19 pandemic. The two interviewers had master’s degrees in health economics (JL) and health-promoting organizational development (HB), were trained in qualitative research and were Ph.D. candidates. Participants were asked to go to a quiet room where they were alone. The participants did not know the researcher, only that they were interested in workplace-related health. This minimized the researchers’ ability to influence the study. Due to the principle of data saturation, the data set was evaluated after the first 15 interviews (single interviews, no repeat interviews). Since there were still open questions about the setting of the inpatient care facilities, additional interview partners were sought. After one more interview, no new information seemed to emerge from the interviews. During all the interviews, field notes were taken and missing demographic data were only asked for after the interview was completed to avoid disrupting the flow of the interview. Interviews were recorded with an audio recording device and professionally transcribed according to Dresing and Pehl (2018).

### 2.5. Data analysis

For analyzing the transcripts, a structuring content analysis following Kuckartz was performed. Internationally, the methodology is very similar to the framework method for analyzing qualitative data (Gale et al., 2013). Data management was carried out using MAXQDA Standard 2020 software from VERBI GmbH, Berlin. Based on a screening of six transcripts, the interview guide, and the topics of interest, a first coding system with main categories was created and discussed by three researchers. Afterward, the interviews were coded according to this coding system. The main codes and text passages were then sighted by a research team of five. Subcategories and characteristics were derived and discussed by this research team. Afterward, the entire data set was completely coded with subcategories and characteristics. The results were not returned to the participants because it was not a participatory evaluation, and the data were analyzed *via* a setting-specific approach.

## 3. Results

The following main categories were deduced: (1) *awareness of WHP activities in the company*, (2) *participation in WHP activities*, (3) *organizational conditions for the participation in WHP activities*, (4) *wishes of the employees*, and (5) *communication and information on the WHP activities*. Each main category was divided into subcategories (refer to Table 3) and then examined for *inhibiting, promoting*, and *explanatory* characteristics.

**Table 3:** Category system with defined main categories and subcategories.

Main category	Subcategories
1. Awareness of WHP activities in the company	Stress related
	Movement oriented
	Nutrition
	Addiction
	Violence related
	Teambuilding activities
	No known WHP activities
2. Participation in WHP activities	Reasons for non-participation
	Reasons for participation
3. Organizational conditions for the participation in WHP activities	Timing
	Distance
	Team-internal agreements
	Work activity/environment
	Workload
	Leadership support
	Corporate culture
	Value health
	COVID-19
4. Wishes of the employees	Content of the WHP activity
	Framework
	Participation
	No interest
5. Communication and information on the WHP activities	Perceived communication channels
	Wishes for communication channels

In the following, the results of the content analysis of the interviews with nurses from acute care hospitals (ACH), inpatient care facility (ICF), and outpatient care service (OCS) are presented in relation to the respective main and subcategories.

### 3.1. Awareness of WHP activities in the company

The main category “*awareness of WHP activities in the company*” included information on WHP activities of the employer on-site, digitally, or in cooperation with external service providers perceived by the employees from their perspective. Seven subcategories were identified: *Stress related, movement oriented, nutrition, addiction related, violence related, team building*, and *no known WHP activities*. A wide range of experiences emerged. Some nurses reported having no WHP activities at all, and some reported having several topic areas at once.

The subcategory *stress related* was defined as WHP activities in the area of stress management and strengthening of mental resources. In this context, companies provided contact persons for problems and confidants, both in the professional context, e.g., in dealing with conflicts but also privately, when emergency childcare was required.

Interviewee (I): “Then there is also a specialist colleague at the company who also helps with problems with psychological disorders or stress or depression or problems with violence. You can go and see her and make an appointment. So you can say that you will get help in any case, that’s the way to go.” (OCS nurse)

In addition, there were activities for optimizing sleep, active relaxation training such as yoga and access to passive relaxation, e.g., through massage activities.

The subcategory *movement oriented* included WHP activities in the area of movement promoting work and physically active employees. It was mentioned that ergonomic work was supported by work equipment and that training in back-friendly working methods, e.g., kinesthetic, also existed. In addition, there were activities for physical balance in the form of exercise courses, e.g., gymnastics and team sports.

The subcategory *nutrition* was defined as WHP activities in the area of healthy nutrition in everyday working life. WHP activities were found in the area of optimization of the on-site nutritional activities, common nutritional activities, and educational activities on healthy nutrition. While the interviewees in ACH were particularly aware of the efforts made by the organizations with regard to cafeteria offerings, those in ICF and OCS were more aware of individual training courses on healthy eating and common cooking activities.

The subcategory *addiction* included information about WHP activities in the area of addiction prevention. There was only one statement in OCS telling about the idea of the company to set up a smoke-quitting WHP activity but no real activity is planned yet.

In the subcategory *violence related*, the most mentioned characteristics were training courses on de-escalation/dealing with violence in the area of ACH and OCS. ICF focused mainly on informal talks with the team or leadership. OCS emphasized this too, next to the training activities. Furthermore, there were structural aids given (emergency button, guidelines for action). Overall settings, there were professional contact persons visible.

I: “Yes, we already have many training courses on the subject [of violence prevention], also many different ones, first of all how best to deal with such a situation. Then also simply further training in which you are shown how to protect yourself, how you can also put the patient out of action in the situation […] without hurting yourself and the patient.” (ACH nurse)

In the subcategory *teambuilding*, activities can be found that serve team-building purposes. Hereby, in ACH, only full-day events for team building were mentioned, thus, it was called “team days.” In ICF, communal eating as a team-building event was mentioned. In OCS, the most versatile activities were mentioned, such as communal eating, parties, meetings, and outgoing activities.

“That’s when we did various team-building activities, had discussions, ate, had a barbecue in the evening, talked about work, that kind of thing.” (ACH nurse)

The subcategory *no known WHP activities* collected statements on not perceiving WHP activities. In all settings, there were some interviewees mentioning not knowing any WHP activity in their company:

Researcher (R): “So what opportunities and offerings do you know of there?” B: “Actually, none at the moment.” (ACH nurse)

### 3.2. Participation in WHP activities

The main category *participation in WHP activities* was defined as statements about the reasons for participation or non-participation in WHP activities. Two subcategories were defined: *reasons for non-participation* and *reasons for participation*. The interviews were searched for inhibiting, promoting, and explaining characteristics.

The subcategory *reasons for non-participation* included reasons for the interviewee not to participate in WHP activities for *organizational* reasons. Overall settings, a long journey to the facility where activities took place was a barrier to participating in WHP activities. For ACH and OCS, time fit to shift work was often an issue. In ACH and OCS, it was mentioned that WHP activities were not suitable for the own work environment (for example, work on a closed station). In ACH and ICF, due to COVID-19, there were not any WHP activities offered. Furthermore, in ACH, it was mentioned that the WHP activities were not attractive for the professions.

“If something takes place where I’m there or right after work, I might attend. But I cannot drive back there and back again after three hours.” (OCS nurse)

“This is just a limited selection and temporally also not suitable for many or for me often not.” (ACH nurse)

The subcategory reasons for participation included reasons for the interviewee to participate in WHP activities for organizational reasons. There were comments by nurses in OCS participating because of the design of the WHP activities. Good communication about the activities and the good affordability were positively assessed. Conspicuous in the expressions of the ICF nurses was the group dynamics they experienced. No comments on this topic were found by nurses working in ACH.

“Most of the offers are also really free or discounted. And I really can’t make any accusations about that. It’s really well communicated and it’s also really financially and everything is possible for every employee.” (ICF nurse)

### 3.3. Organizational conditions for participation in WHP activities

The main category *organizational conditions for participation in WHP activities* was defined as the general conditions in the context of work that play a role in participation in WHP activities. Nine subcategories were defined: *timing, distance, team-internal arrangements, work activity/environment, workload, leadership support, corporate culture, value health and COVID-19*, and *others*. The interviews were analyzed on inhibiting and promoting characteristics.

The subcategory *timing* described the interviewees’ perception of how the noticed WHP activities can be used in the context of shift work. It was separated into *promoting* and *inhibitory* factors. Participating in a WHP activity during work hours was mentioned as promoting factor in ICF and OCS. In all interviewees from OCS and ACH, it was found inhibiting that the scheduling of the WHP activities was not compatible with shift work. There were no findings for ICF in this regard.

“But the problem is, these are often series events where you then have to sign up for all of the appointments. These appointments are often not compatible with nursing.” (ACH nurse)

The subcategory distance described the interviewees’ perception of the role of spatial distance in the participation of WHP activities. In both the settings ACH and OCS, nurses reported that a large distance between the WHP activities and the place of work inhibited participation if the WHP activity was scheduled differently from the shift work.

“[…] I live 30 kilometres away. And if something takes place […] where I’m there or right after work, I could participate. But I can’t drive there again after three hours and come back. If I live in the city, I would, I could also come back.” (OCS nurse)

No statements were given on inhibitory factors by nurses in ICF. Overall settings, no promoting factors for participation in WHP activities due to a large distance were found.

The subcategory workload described how the amount of work or understaffing influenced participation in WHP activities. It was separated into promoting and inhibitory factors. Only inhibiting factors were found for this topic. Across all settings, the workload was clearly reported to be very heavy. This was due to more and more multimorbid patients, a higher documentation workload and understaffing for various reasons. In addition to the negative influence of this on personal health behavior and the organization of breaks, interviewees explicitly reported that this inhibited any health activity after the end of the shift due to stress. With regard to participation in WHP activities, a nurse in ICF made the explicit reference that participation in WHP activities was inhibited due to the highly demanding workload and a shortage of staff.

“when I do a shift that I now do with two colleagues, when I do it with five or six colleagues. And everyone can really go about their work in a relaxed manner. And at the end of the day, they are not completely exhausted and somehow only want to go home. Then he or she would definitely spend another two hours in the back course.” (ICF nurse).

The subcategory leadership support described the influence of the manager on nurses in the participation in WHP activities. It was separated into promoting and inhibitory factors. In the setting of ACH and OCS, some experiences became visible where employees experienced that their leadership affirmed health-promoting behavior. The focus in this subcategory yet laid on the promotion of WHP activities by leadership. Among all settings, there were some experiences both on leadership support for participation and some not experiencing that.

“[…] We sit together every 14 days and see if there is anything new. And if there are new activities, then I’m informed that there are” (ACH nurse)

“R: […] how is the issue of employee health addressed by your direct leadership? I: Not at all.” (OCS nurse)

The subcategory corporate culture described the attitude that prevailed in the company at various levels of the members. The results varied among the settings. Some positive comments were found regarding the general corporate culture. The interviewees reported that despite the staff shortage, they felt that they were being looked after. It was mentioned that there was a family atmosphere and that there was understanding and open communication, especially in the case of OCS.

“That is a very big factor, they always take care, they always make sure that everyone is doing well.” (ICF nurse)

Good job security and satisfaction with the workplace and the structures were also mentioned across all settings but not in every interview. Some interviewees reported that there was a good sense of community and that the atmosphere was characterized by trust. In some cases, it was reported that there was a clear sense of humanity in the hospital despite the economic pressure.

“That is certainly the case, that, the team spirit is very, very big. And as much as the (hospital name), the management level has to think economically, yes, but despite that, people still play a big role.” (ACH nurse)

Other experiences from the hospital were a miserable atmosphere, a perception of replaceability, and a lack of appreciation from the employer.

“[…] a colleague says I’m quitting because I can’t do it anymore, they don’t ask what we can do to get you to stay. […] On the contrary, one is then offended that he leaves. […] I’m speaking not just about my clinic, I think it’s the same in other large clinics.” (ACH nurse)

Some ICF and ACH nurses described that, after all, they only associated negativity, stress, and pressure with the employer.

“I think if I had the feeling: “I have to get out of here and I want to go home,” I wouldn’t do a back course in the hospital afterwards. Because afterwards I think, “He still wants something from me or he’s talking about work or something else. I think there would be strategically more favourable possibilities.” (ACH nurse)

The subcategory value health described the perceived anchoring of the value of health in the corporate philosophy. Here, there were very different characteristics across all settings. It went from a clear perceptibility of the value of health in the company to only a superficial presentation where this value is not visible.

“I rate the [importance of employee health] as not very high.” (ACH nurse)

“Should you ever have to cover, you have a compensation day directly next week. […] in any case, health is very important here.” (ICF nurse)

The subcategory COVID-19 collected information on the influence of COVID-19 on WHP activities in the facility. Overall settings, it became visible that COVID-19 had an inhibiting influence on health-promoting activities, and the opportunities for digitalization were partially missed. In some cases, the facilities slowly started activities again.

Other comments that appeared in this main category were the mention of a problematic structure for nutritional services and too few places for participation in education courses in the hospital. Across all settings, comments were made that no real breaks could be taken. In addition, it was noted by ACH and ICF nurses that WHP activities may not have received much recognition.

“Yes, I also don’t know whether that would necessarily be appreciated if there was somehow a training program for back health or something like that.” (ICF nurse)

### 3.4. Employee wishes for WHP activities

The main category *employee wishes* included the desirable design of the WHP activity from the point of view of the interviewees. Four subcategories were defined: *content, framework, participation*, and *no interest*. The subcategories, then, were searched for inhibiting, promoting, and explaining characteristics.

The subcategory *content* collected wishes on the design of the content. As far as the desired topics are concerned, across all settings, WHP activities were desired in the area of smoking prevention, exercise (ergonomic work, support for aids, and exercise activities/physiotherapeutic support), nutrition activities, de-escalation training, mental activities (supervision, relaxation training), and team-building activities (outdoors). Partly behavior-oriented activities were desired, but partly improved structures were also desired, such as more participation places in de-escalation courses, improved infrastructure in the catering activities, and provision of aids.

“For example, I would like to have physiotherapy. Doing exercises. Rhythmic gymnastics, a little dance, a little different. For ten minutes, for fifteen minutes, that we move a little bit, laugh a little bit.” (ICF nurse)

Regarding the content of the WHP activities, there were no specific topic requests for WHP activities for the hospital nurses. In OCS, the nurses wished for group activities and events in person instead of digitally. In ICF, the nurses expressed the desire for WHP activities to be fun and useful.

“And the fun factor is also important, yes. You should make the offer for the sake of wanting to and not for the sake of having to, yes.” (ICF nurse)

The subcategory *framework* was defined as the design of the framework of the WHP activity (type, scope, and series of appointments). In this context, the desire for more numerous, more flexible WHP activities adapted to shift work became clear across all settings. The wishes for the localization of the WHP activities were not uniform, as some wanted it at the workplace and others explicitly not at the place of work. Ideas for enabling a WHP activity participation were the use of a rotation principle or the request of possible time periods. In OCS, the integration of WHP activities into working hours or compensatory time off was mentioned. The idea for discounts on work clothes and external health care providers such as gyms was also mentioned in all settings. Good communication of WHP activities and more slots to participate in de-escalation training were addressed in one hospital.The subcategory *participation* described wishes concerning the participatory design of WHP activities. In hospitals, the wish for asking around for suitable time slots was mentioned.

“That you simply get an opinion from the hospital, from all colleagues […]“Okay, this and this and this are the times […] I would like to participate in that and I could also participate in that”. And then to see: “Okay, which are the times that are mentioned most often and what kind of possibilities do we have to implement that?.” (ACH nurse)

The subcategory *no interest* collected information on interviewees not having an interest in WHP activities and therefore not having any wishes. Comments on not wanting to stay longer at work, not being motivated to participate, and seeing a deeper source of the problems were found in OCS and ICF.

R: “Which topics would you be more interested in?”

I: ‘‘End of work (laughter). I don’t have any desires there, or I don’t lack anything. When my shift is done, then you can’t attract me with anything anymore, I think.” (ICF nurse)

### 3.5. Communication and information on the WHP activities

The main category c*ommunication and information of the WHP activities* described communication channels through which the WHP activities were perceived or employees could obtain information in the company. This is divided into two subcategories: *perceived communication channels* and *wishes for communication channels*.

The subcategory *perceived communication channels* described the communication channels and information platforms on which WHP activities were noticed. Here, the communication of the WHP activities *via* Whatsapp, email, notices, and the personal or telephone approach was perceived across all settings. Furthermore, in the OCS setting, information was handed over *via* the inbox or a company app. In the ACH, the intranet and a training booklet were named communication media.

Overall, in all settings, the posting of notices and receiving emails were explicitly positively evaluated.

“So, I have to say, that’s really very transparent and very positive. And everyone can really see it. […] in the offices, in the info points, things are really regularly posted everywhere on well-designed posters. Emails are sent, really with the dates and the telephone numbers.” (ICF nurse)

Personal communication and the use of communication apps in ICF and OCS were explicitly rated positively. In ICF, team meetings and information *via* telephone were considered positive.

“However, there were also cases where employees did not read all emails or not all information was shared in the intranet: “So, I know that not all employees read any emails.” (ACH nurse)

The subcategory wishes for communication channels collects information on how wishes and ideas on WHP activities could be better communicated. Here, it appears that OCS wished for written communication *via* email or notice board. ICF also favored emails and direct personal communication. In the hospital, the widest range of favored communication channels was mentioned (notice board, team meeting emails, and personal communication).

“I think it would be good/. Also the people who offer such an activity[…] that these people simply go into the team meeting and say: ‘‘I offer this and that. This is what it looks like.’’[…]. I think that would achieve even more when you go into these team meetings. But a personal approach is always better than an e-mail.” (ACH nurse)

## 4. Discussion

### 4.1. Summary of findings

The aim of this qualitative study was to identify organizational factors that promote or inhibit participation in WHP activities from the perspective of nurses. The results show that there is a wide range of barriers and facilitators at the organizational level, some overall settings, and others setting-specific. The high workload and the fitting of WHP activities with shift times are particularly striking.

The type and frequency of WHP activities vary widely across settings, from none offered at all to many offered. Relaxation training, ergonomic training, sports courses, improved nutrition services, nutrition courses, training courses on de-escalating violence, professional contact person for violent incidents, and different team-building events. In OCS, good communication, good affordability, and the design of the WHP activities are stated as facilitating participation in WHP activities. OCS nurses like the group feeling. Participating during work time promotes participation in OCS and ICF. For ACH and OCS nurses, a large distance between home and the WHP activities in regard to the different shift times is an inhibiting issue. The wish for numerous, flexible WHP activities becomes clear. High workload overall settings are stated to inhibit as well as a negative association with the employer in ICF and ACH. With regard to the communication of WHP activities, the sending of emails and posting of notices are desired and positively evaluated across all settings.

### 4.2. The study aims at the context

In our study, the type of perceived WHP activities varies greatly across all settings from no activities at all to several different activities. Active/passive relaxation training, ergonomic/sports training, nutrition activities, activities for de-escalating violence, and professional and different team-building events are named as existing. There are wishes for implementing smoking prevention and activities in the common topics of exercise (ergonomic work, support for aids, and exercise activities/physiotherapeutic support), nutrition activities, de-escalation training, mental activities (supervision, relaxation training), and team-building activities (outdoors).

A German study on nurses points attention to a topic not found in our study: communication training (Ehegartner et al., 2020). Reduction of stress, recovery, and solving conflicts yet are announced here too (Mojtahedzadeh et al., 2021). For German nurses, practical preventive measures are required primarily in the areas of back health, strengthening, and again relaxation (Ehegartner et al., 2020). The diversity of interests is perhaps less about settings and more about whether it addresses behaviors nurses care about (Hammerback et al., 2015). In any case, in our study, the wish for numerous, flexible WHP activities becomes clear. In other professions, it was found that a broad array of program activities can raise participation levels in WHP (Robroek et al., 2009).

With regard to framework conditions, good communication, good affordability, and the design of the WHP activities facilitate participation in WHP activities.

Nurses of outpatient care service (OCS) seem to like the group feeling. For ACH and OCS nurses, a large distance between home and the WHP activities in regard to the different shift times is an inhibiting issue. The literature confirms the fundamental challenge of different working hours in the shift system and the work outside the company for WHP in OCS. It seems difficult to design activities in such a way that they can be used equally by all employees (Neumann et al., 2022). Another United States study on nurses emphasizes this finding for the setting of ICF. Difficulty with a time release, making time, and scheduling issues appear to be influencing participation rates (Zhang et al., 2016).

The goal of fulfilling the healthcare mandate seems to have a structural dominance over other long-term goals, such as maintaining the health and working ability of nurses over all settings (Krupp et al., 2020). In our study, participating during work time was promoting participation in OCS and ICF. United States study results support this finding, as release time for participation and management support are identified as the most important factors for WHP in the nursing setting (Havermans et al., 2016; Zhang et al., 2016). In our study, management support is partly experienced, but it is subjectively not brought in connection with the participation, so there is still an open question here. However, the United States study on nurses finds that support from multiple levels such as managers seems to be important for participation in WHP activities for nurses (Zhang et al., 2016). In other professions, it is even stated to be the most influencing factor (Rojatz et al., 2015).

The reason why manager support and WHP are not brought together here may have methodological reasons. In our qualitative study, it may be that the nurses are not that aware of the connection between WHP and the promotion of WHP by their managers. However, this could perhaps be measured quantitatively as is the case in other studies. Nevertheless, there is further research needed on how managers can support their teams by communicating and promoting WHP topics.

The topic “high workload” is stated as inhibiting participation in WHP activities overall settings as well as a negative association with the employer in ICF and ACH. Other results from Germany show that a high work density with a simultaneous shortage of nurses often pushes the implementation of WHP activities into the background (Krupp et al., 2020). In terms of the quality of association with the employer, other international nursing studies find that participation is significantly associated (*p* < 0.05) with higher satisfaction with the job, work, lower stress, exhaustion, and cynicism (Ledikwe et al., 2018).

In the literature, there are other influencing factors for WHP in nursing that have not been mentioned by our interviewees or have not been put into a subjective context. Positive influence on ICF nurses can be found in employee awareness, engagement, and WHP being integrated into everyday organizational structures by the top management. Furthermore, a participatory culture, providing financial resources and the presence of a functional committee to promote good communication and motivate employee participation, seems to be facilitating (Zhang et al., 2016). Next to the establishment of a health committee, administrative support and integration of activities in the organization are found to be facilitating for healthcare workers (Ledikwe et al., 2018). The “patient” priority and a limited appreciation of their own wellbeing are barriers for healthcare workers (Ledikwe et al., 2017). In other professions, organizational structures, available resources, reorganizations, the presence of multiple company locations, and a poor psychosocial environment are listed as influencing factors for WHP (Rojatz et al., 2015).

With regard to the communication of WHP activities, ICF and OCS experience mostly personal communication and communication *via* apps, ICF team meetings, and telephone contact. ACH mentions communication *via* intranet and having a training booklet. The sending of emails and posting of notices are desired and positively evaluated across all settings. A study on ICF nurses reports that a lack of communication is experienced as a barrier to participation in WHP. Employees and middle managers report frustration with the team members primarily talking to each other, rather than trying to get more front-line staff involved (although they think they do). A German qualitative study enhances the enabling of participation and communication and the creation of transparency for the success of WHP (Brand et al., 2017).

### 4.3. Strengths and limitations

The results of our study provide insight into which organizational factors may be promoting and inhibiting participation in WHP activities from the perspective of nurses. This creates a basis not only for initial adjustments in practice but also for in-depth research to explore further interrelationships. Despite the new insights presented, the study’s weaknesses must also be pointed out. As our participants were selected through contacts in care organizations who participated in the WHP-BAGGer project, this can be a sample that is already better positioned than other care facilities. With regard to the age of the participants, it can be seen that no interviewee was younger than 25 years (the lower limit of the sample was 18 years). This could have several reasons. Possibly, these persons were not interested in participating in an interview study or think, they don’t have enough experience to talk about this topic in an interview. However, due to the fact that the contact person at the facility initiated contact with potential interview partners, we do not have any concrete information on this. The interviews were conducted by telephone, which had the advantage that the participants were in a familiar environment. Nevertheless, an audio-only track conveys less information about the interviewee than a face-to-face conversation. Interview fatigue could have played a role. We always recorded possible observations during the interview and could not detect any signs of fatigue. In fact, the interviewees were very talkative. The data saturation was stated as no more new topics (code saturation) were mentioned, but it is still questionable whether full meaning saturation was achieved, as some questions could have used more depth (Hennink et al., 2017). In terms of other methods, it would have been interesting to examine an exchange of perspectives, such as a focus group, between people who are involved in the management of WHP and the target group’s reasons for or against participation. Furthermore, an expansion of quantitative research on the basis of a large sample on these topics would be interesting to test for significant correlations.

At the present time, health insurers and employers are interested in expanding WHP due to positive indications for it. Employees are also interested in WHP activities, but participation rates are low. Our qualitative results provide information on what can be useful to increase participation. A meta-analysis in other professions indicates larger intervention effects among workers with higher program compliance, which emphasizes the importance of sustained participation with regard to effectiveness (Coenen et al., 2020). Designing WHP activities deserves attention to achieve a better insight into what works for whom in which context and to make sure that successful WHP programs are sustainable in practice (Robroek et al., 2021). To develop this, structured process evaluations to monitor the implementation alongside effect evaluations are needed (Havermans et al., 2016).

## Data availability statement

The data presented in this study are available on request from the corresponding author.

## Ethics statement

The studies involving human participants were reviewed and approved by German Ethics Committee of the German Sport University Cologne (reference numbers no. 050/2021). The patients/participants provided their written informed consent to participate in this study.

## Author contributions

HB, JL, and AS: conceptualization and methodology. HB: formal analysis, writing—original draft, and visualization. HB and JL: investigation. AS: resources, funding acquisition, and supervision. JL and AS: writing—review and editing. HB and AS: project administration. All authors contributed to the article and approved the submitted version.

## Funding

This research was funded by the Federal Ministry of Health (BMG), grant number “BVA 2520ZPK744.” This study belongs to the BAGGer project (workplace offers for health promotion and violence prevention in WHM: impact model-based conception and evaluation of a WHP program).

## Conflict of interest

The authors declare that the research was conducted in the absence of any commercial or financial relationships that could be construed as a potential conflict of interest.

## Publisher’s note

All claims expressed in this article are solely those of the authors and do not necessarily represent those of their affiliated organizations, or those of the publisher, the editors and the reviewers. Any product that may be evaluated in this article, or claim that may be made by its manufacturer, is not guaranteed or endorsed by the publisher.
